# Endothelial biology and ATP-sensitive potassium channels

**DOI:** 10.1080/19336950.2017.1412151

**Published:** 2018-01-02

**Authors:** Qadeer Aziz, Yiwen Li, Andrew Tinker

**Affiliations:** William Harvey Heart Centre, Barts & The London School of Medicine & Dentistry, Queen Mary, University of London, Charterhouse Square, London, UK

**Keywords:** ATP-sensitive, potassium channel, endothelium, coronary circulation, hypoxia, adenosine receptor, ischaemia-reperfusion injury

ATP sensitive potassium (K_ATP_) channels are metabolic sensors with channel activity promoted by decreases in ATP and\or increases in MgADP. The most studied are channels present in cardiac myocytes and pancreatic β cells but K_ATP_ currents also exist more widely in a range of tissues and organs [1,2]. The channel is a hetero-octamer comprising four sulphonylurea receptors and four pore-forming inwardly rectifying subunits (either Kir6.2 or Kir6.1). Kir6.2 underlies many of the traditional K_ATP_ channel populations. Kir6.1 is less studied and thought to underlie the current in vascular smooth muscle cells, however it appears to be ubiquitously expressed [1,^3^]. K_ATP_ currents are also present in endothelial cells but defining their physiological role is difficult; pharmacological tools and global knockout mice will also lead to effects on vascular smooth muscle and nerve endings [4]. To overcome this limitation we have used cre\loxP technology to selectively delete the K_ATP_ channel subunit Kir6.1 in endothelium (eKO) and studied the phenotype of the mice [5]. The deletion of this subunit was indeed sufficient to abolish expression of K_ATP_ currents in endothelial cells when studied using patch-clamp [5].

**Figure 1.**
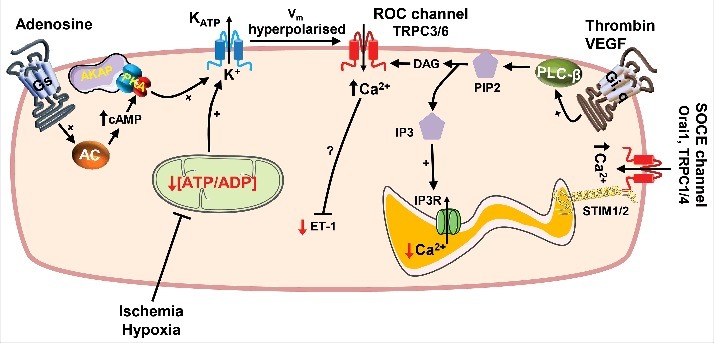
A figure illustrating the various pathways for calcium mobilisation in endothelial cells and the relationship to potential K_ATP_ channel activation.

Intracellular calcium plays an important role in endothelial function by being linked to mediator release and is also important for angiogenesis. Unlike muscle cells the pathway for membrane calcium entry is not mediated by voltage-dependent calcium channels but by TRP channels (including TRPC1/3/4/6 and TRPV4) and the Orai\STIM1 complex [6]. Thus calcium entry into endothelial cells is determined by the electrochemical gradient and as result, hyperpolarisation increases calcium entry. A number of pathways, in addition to membrane potential can increase calcium entry, and these include store and receptor-operated calcium entry. The receptors are coupled to the G_q\11_ family of G-protein and include muscarinic, protease-activated and bradykinin receptors. Cytosolic endothelial calcium is also significantly influenced by IP_3_ mediated release from intracellular stores. Thus opening K_ATP_ channels could potentially promote calcium entry as illustrated in Figure 1. Indeed this was the case when measuring the effect of potassium channel openers and K_ATP_ channel inhibitors on calcium concentration in endothelial cells present on aortic valve leaflets. These drug effects were abolished in the eKO mice. We then examined the role of the endothelial K_ATP_ channel in coronary artery reactivity. In eKO mice basal coronary perfusion pressure was not, elevated but vasorelaxation was impaired during hypoxia. Furthermore, cardiac ischaemia-reperfusion injury was increased in eKO mice compared to littermate controls. Thus it is clear that the endothelial channel contributes to the vascular response to hypoxia. In fact when compared to a murine line in which K_ATP_ channels were deleted from vascular smooth cells the magnitude of the fall in coronary perfusion pressure during hypoxia and that of infarct size after ischaemia reperfusion injury were at least comparable and possibly larger.

A critical question is how is the metabolic signal sensed and transduced by the endothelial cells. We explored one possibility, namely that release of adenosine from ischaemic tissues may bind to the adenosine family of G-protein coupled receptor. It has been established that K_ATP_ channels containing Kir6.1 are activated by protein kinase A through direct channel phosphorylation and this is important for the action of vasodilators in smooth muscle cells [5]. The A2 family of adenosine G-protein coupled receptor are known to couple to the stimulatory G-protein and thus stimulate adenylate cyclase, increase cAMP and activate protein kinase A (PKA)[7]. With this in mind we examined whether K_ATP_ currents could be activated in endothelial cells by an adenosine receptor agonist and indeed found this to be the case. In contrast, in eKO mice this did not occur. K_ATP_ channels are also directly sensitive to metabolism and this could contribute to the response. Although metabolic sensitivity can be demonstrated for Kir6.1 containing channels, we have viewed them as more prominently regulated by hormonal cellular signalling pathways [1].

Is there more to be learnt about the role of the endothelial K_ATP_ channel? K_ATP_ channels can clearly influence endothelial cell membrane potential sufficiently to lead to membrane hyperpolarisation and calcium entry. Thus there is the potential for them to be involved in modulating endothelial cell biology more broadly. For example, intracellular calcium in endothelial cells is important for barrier function, immune surveillance for pathogens and angiogenesis [6]. There are some interesting data showing that in addition to influencing endothelial mediator release, the expression of endothelin (ET-1) may also be reduced by potassium channel opening drugs [8]. In addition, it would be informative to systematically characterise all G-protein coupled receptor pathways coupled to the stimulatory G-protein that might regulate K_ATP_ channels as this mechanism could contribute to a range of physiological processes. A second issue is what influence the endothelial channel more widely has in the vasculature. For example, does it shape resting blood pressure in some way or act to prevent endothelial dysfunction? These are all questions that can be pursued using murine models with conditional endothelial deletion of K_ATP_ channel subunits.
